# HIV-1 Superinfection Occurs Less Frequently Than Initial Infection in a Cohort of High-Risk Kenyan Women

**DOI:** 10.1371/journal.ppat.1003593

**Published:** 2013-08-29

**Authors:** Keshet Ronen, Connor O. McCoy, Frederick A. Matsen, David F. Boyd, Sandra Emery, Katherine Odem-Davis, Walter Jaoko, Kishor Mandaliya, R. Scott McClelland, Barbra A. Richardson, Julie Overbaugh

**Affiliations:** 1 Human Biology Division, Fred Hutchinson Cancer Research Center, Seattle, Washington, United States of America; 2 Public Health Sciences Division, Fred Hutchinson Cancer Research Center, Seattle, Washington, United States of America; 3 Department of Medical Microbiology, University of Nairobi, Kenyatta National Hospital, Nairobi, Kenya; 4 Coast Provincial General Hospital, Women's Health Project, Mombasa, Kenya; 5 Department of Global Health, University of Washington, Seattle, Washington, United States of America; 6 Vaccine and Infectious Disease Division, Fred Hutchinson Cancer Research Center, Seattle, Washington, United States of America; 7 Department of Biostatistics, University of Washington, Seattle, Washington, United States of America; University of Miami, United States of America

## Abstract

HIV superinfection (reinfection) has been reported in several settings, but no study has been designed and powered to rigorously compare its incidence to that of initial infection. Determining whether HIV infection reduces the risk of superinfection is critical to understanding whether an immune response to natural HIV infection is protective. This study compares the incidence of initial infection and superinfection in a prospective seroincident cohort of high-risk women in Mombasa, Kenya. A next-generation sequencing-based pipeline was developed to screen 129 women for superinfection. Longitudinal plasma samples at <6 months, >2 years and one intervening time after initial HIV infection were analyzed. Amplicons in three genome regions were sequenced and a median of 901 sequences obtained per gene per timepoint. Phylogenetic evidence of polyphyly, confirmed by pairwise distance analysis, defined superinfection. Superinfection timing was determined by sequencing virus from intervening timepoints. These data were combined with published data from 17 additional women in the same cohort, totaling 146 women screened. Twenty-one cases of superinfection were identified for an estimated incidence rate of 2.61 per 100 person-years (pys). The incidence rate of initial infection among 1910 women in the same cohort was 5.75 per 100pys. Andersen-Gill proportional hazards models were used to compare incidences, adjusting for covariates known to influence HIV susceptibility in this cohort. Superinfection incidence was significantly lower than initial infection incidence, with a hazard ratio of 0.47 (CI 0.29–0.75, p = 0.0019). This lower incidence of superinfection was only observed >6 months after initial infection. This is the first adequately powered study to report that HIV infection reduces the risk of reinfection, raising the possibility that immune responses to natural infection are partially protective. The observation that superinfection risk changes with time implies a window of protection that coincides with the maturation of HIV-specific immunity.

## Author Summary

HIV-infected individuals with continued exposure are at risk of acquiring a second infection, a process known as superinfection. Superinfection has been reported in various at-risk populations, but how frequently it occurs remains unclear. Determining the frequency of superinfection compared with initial infection can help clarify whether the immune response developed against HIV can protect from reinfection – critical information for understanding whether such responses should guide HIV vaccine design. In this study, we developed a sensitive high-throughput method to identify superinfection and used this to conduct a screen for superinfection in 146 women in a high-risk cohort. This enabled us to determine if first HIV infection affects the risk of second infection by comparing the incidence of superinfection in this group to the incidence of initial infection in 1910 women in the larger cohort. We found that the incidence of superinfection was approximately half that of initial infection after controlling for behavioral and clinical differences that might affect infection risk. These results suggest that the immune response elicited in natural HIV infection may provide partial protection against subsequent infection and indicate the setting of superinfection may shed light on the features of a protective immune response and inform vaccine design.

## Introduction

Development of a safe and effective prophylactic HIV vaccine remains enormously challenging, due to the virus's high diversity and our limited understanding of immune correlates of protection. While most effective vaccines are designed to mimic natural infection and protective immune responses to it, such a template for HIV vaccine design remains elusive, since sterilizing immune responses to natural infection have not been observed. A priority of HIV vaccine development is, therefore, to identify settings where natural infection elicits some immune functions desired in a vaccine. For example, HIV-infected individuals who spontaneously control viral replication have provided insights into immune mechanisms of HIV control [1]. However, models where the response, rather than delaying disease, prevents infection – the ultimate goal of a prophylactic vaccine – remain less well characterized. Studies of superinfection (reinfection from a different partner) provide a unique model in which to investigate the impact of pre-existing responses on susceptibility to infection by diverse circulating viral variants, which include multiple subtypes with up to 30% sequence variation.

HIV superinfection has been reported in a number of settings [2]–[13], implying that HIV acquisition can occur despite the immune response to initial infection. However, it remains an open question whether pre-existing infection affords some protection from superinfection, and individuals who do become superinfected are a select subset deficient in a particular aspect of immunity. Published estimates of superinfection incidence vary from no identified cases [1], [14]–[16] to rates roughly similar to initial infection [2]–[13], [17], [18]. These discrepancies are largely explained by differences in participant inclusion criteria and study design. The studies that have directly compared initial and superinfection incidence have had limited statistical power due to cohort size [5], [12], [17], [18] or number of cases of superinfection identified [3], [8]. Additionally, methods used to identify superinfection have evolved. Superinfection is most reliably detected in longitudinal samples by the presence of a single viral clade initially followed by introduction of a second phylogenetically distinct clade [19]. Detection sensitivity is dependent on the number of genomic regions analyzed [12], as well as sequencing depth [20]. Until recently, sequences were obtained by limiting dilution amplification and Sanger sequencing [5], [6], [12], [17], which limits detection to cases where the second virus is relatively abundant. The development of next generation sequencing (NGS) has enabled higher-throughput, deeper sequencing of large cohorts [20], [21].

To date, the largest study to examine the rate of superinfection in a prospective seroincident cohort was a NGS screen by Redd *et al.* of 149 individuals in which 7 cases were identified [8]. No statistically significant difference was found between the incidences of initial infection and superinfection, though the relatively small number of cases may have resulted in limited statistical power. A greater number of cases was found in a high-risk cohort in Mombasa, Kenya, with 12 cases of 56 women screened [5], [12], [17]. However, this study used Sanger sequencing to sample ∼7 clones per sample, which could miss lower frequency variants, and was not powered to compare incidences. In the present study, we developed a NGS method for identification of superinfection, and used it to screen 129 women in the same Mombasa cohort, including those classified as singly infected in the prior study. We identified 9 additional cases of superinfection, for a total of 21 cases in this cohort. These combined data enabled comparison of the incidence rates of initial infection and superinfection.

## Results

### Identification of superinfection cases in a NGS screen

**Figure 1:**
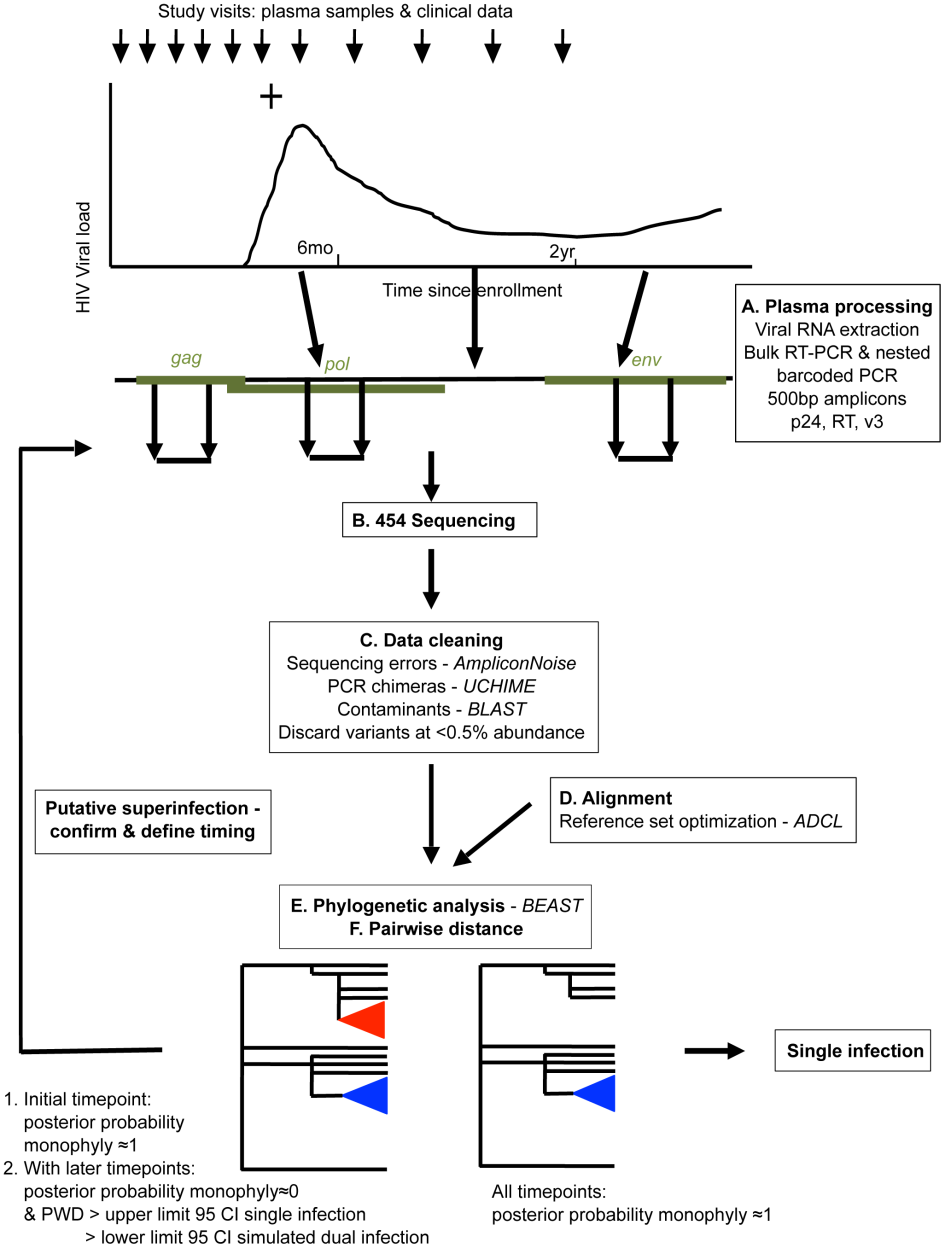
Overview of NGS superinfection screening pipeline.

In order to conduct a sensitive, high-throughput screen for superinfection in the Mombasa cohort, we developed a pipeline for amplification, next-generation sequencing (NGS), data cleaning, and phylogenetic and sequence diversity analysis of longitudinal plasma RNA (Fig. 1). One-hundred thirty-two women met our selection criteria for the NGS superinfection screen, with a median follow-up time of 2046 days (IQR 1265–2848). We successfully amplified *gag*, *pol* and *env* at three timepoints in 115 women and at least two genomic regions in at least the first and last timepoints in 129. The remaining 3 women were dropped from analysis. In total, ∼1.7 million raw sequencing reads were obtained, with ∼1.25 million passing quality filtering: a median of 901 per amplicon per sample.

Women were considered putative superinfection cases if the posterior probability of monophyly supported single infection at the earliest studied timepoint followed by introduction of a distinct viral clade and increased viral diversity consistent with that seen in simulated dual infection (Fig. 1e&f). Putative cases of superinfection were confirmed and their timing specified by analyzing intervening timepoints. Nine cases of superinfection were detected and their timing specified. One case of suspected dual infection was detected, in which two clades were detected at the earliest sample analyzed (60 days post-infection (dpi)) and throughout infection (data not shown).

### Characteristics of superinfection cases

**Figure 2:**
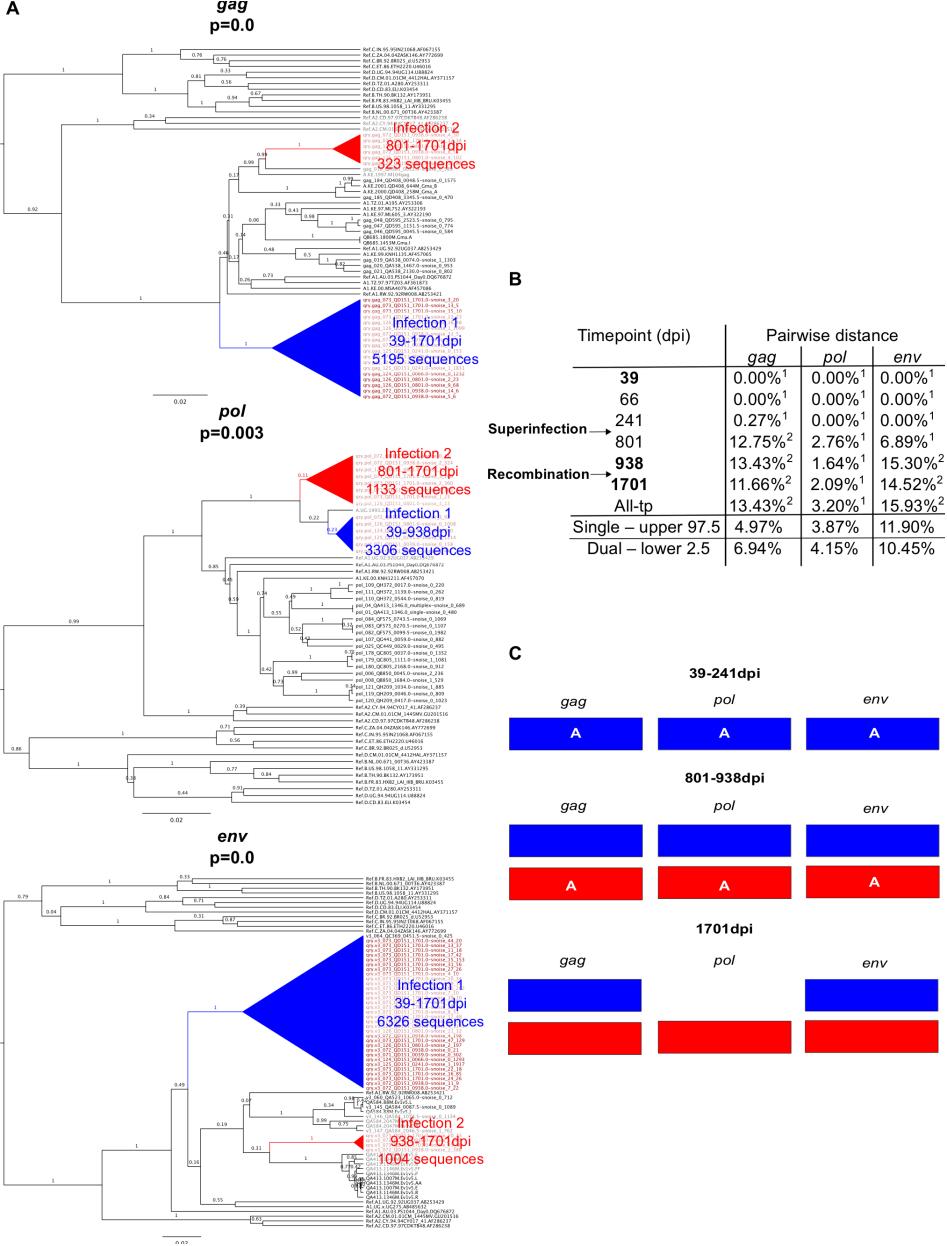
Superinfection case QD151. **A.** Phylogenetic trees representing viral sequence from all timepoints in each genomic region analyzed. The corresponding posterior probability of monophyly is displayed. Initial variant branches are collapsed and highlighted in blue, superinfecting variant branches in red. Red branch labels mark query sequences from individual QD151 at all timepoints, black labels mark reference sequences. **B.** Maximum percent pairwise distance (PWD) between sequences within each timepoint listed or across all timepoints (All-tp). The 97.5% confidence limit of the distances observed among viral sequences within known singly infected individuals and the 2.5% confidence limit within simulated mixtures of sequences from two individuals are shown for comparison. Timepoints shown in bold type are the 3 samples originally screened; the rest were subsequently sequenced to specify superinfection timing. ^1^ PWD within 95% confidence interval observed for single infection. ^2^ PWD outside 95% confidence interval for single infection and within 95% confidence interval for dual infection. **C.** Schematic summarizing viral variants detected in each genomic region over time (initial variant depicted in blue, superinfecting variant in red). Viral subtype is indicated.

**Figure 3:**
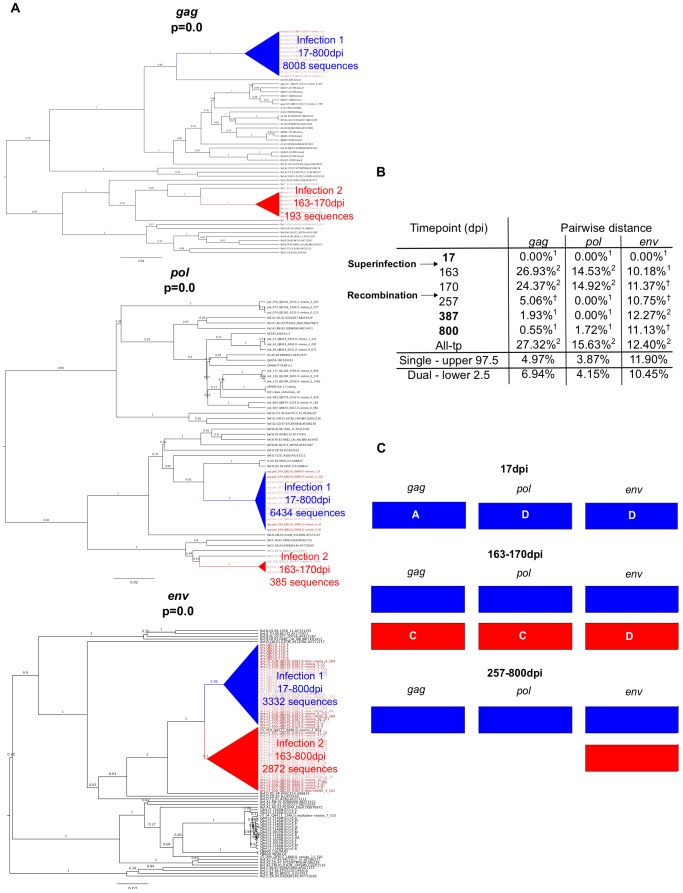
Superinfection case QB210. **A.** Phylogenetic trees representing viral sequence from all timepoints in each genomic region analyzed. The corresponding posterior probability of monophyly is displayed. Initial variant branches are collapsed and highlighted in blue, superinfecting variant branches in red. Red branch labels mark query sequences from individual QB210 at all timepoints, black labels mark reference sequences. **B.** Maximum percent pairwise distance (PWD) between sequences within each timepoint listed or across all timepoints (All-tp). The 97.5% confidence limit of the distances observed among viral sequences within known singly infected individuals and the 2.5% confidence limit within simulated mixtures of sequences from two individuals are shown for comparison. Timepoints shown in bold type are the 3 samples originally screened; the rest were subsequently sequenced to specify superinfection timing. ^1^ PWD within 95% confidence interval observed for single infection. ^2^ PWD outside 95% confidence interval for single infection and within 95% confidence interval for dual infection. † PWD within 95% confidence intervals observed in both single and dual infection. **C.** Schematic summarizing viral variants detected in each genomic region over time (initial variant depicted in blue, superinfecting variant in red). Viral subtype is indicated.

Example data from two cases of superinfection are summarized in Figures 2 and 3. Initial screening of subject QD151 (Fig. 2) showed monophyletic subtype A infection at 39 dpi and two subtype A clades in all three genes at 938 and 1701 dpi. In subsequent analysis of intervening timepoints the second clade was first detectable at 801 dpi (Fig. 2a). At this time, pairwise distance increased sharply, for example in *gag* from 0.27% at 241 dpi, to 12.75% at 801 dpi (Fig. 2b), into the range observed in simulated dual infections. These observations supported introduction of a second subtype A variant between 241 and 801 dpi. The initial clade was no longer detectable in *pol* at 1701 dpi, suggestive of a genomic recombination event (Fig. 2c). Similarly, subject QB210 (Fig. 3) showed initially monophyletic infection with a subtype A/D virus, followed by introduction of a subtype C/D virus at 163 dpi, evidenced by polyphyly and a shift in pairwise distance (>10%) in all 3 genes (Fig. 3a and 3b). In intervening timepoints, the second variant could be detected in all genes at 163–170 dpi, but was undetectable in *gag* and *pol* after 170 dpi, indicating recombination (Fig. 3c).

Characteristics of the 9 new cases of superinfection are summarized in Table 1 and Figure S2. In all but two cases the superinfecting variant was detected in all 3 amplicons in at least one timepoint. In all cases, the superinfecting variant was detected at multiple timepoints in at least one amplicon. In one case (QC369), the initial variant became undetectable in any amplicon following superinfection, suggesting it was replaced, to our detection limit, by the superinfecting variant. Both variants were detected at two timepoints each, the initial variant at 17 dpi and 28 dpi, and the superinfecting variant at 143 dpi and 451 dpi (Fig. S2), indicating this result was not due to contamination. Further, the possibility of sample mix-up was excluded by HLA-typing (data not shown). As illustrated in Figures 2, 3 and S2, in the other 8 cases, variants were intermittently detected in different amplicons at different times, suggestive of genomic recombination and dynamic turnover of the circulating viral population.

**Table 1:** Summary of 9 new superinfection cases in the Mombasa cohort.

ID	Window SI [midpoint] (dpi)	Initial subtype			SI subtype			Virus outcome
		*gag*	*pol*	*env*	*gag*	*pol*	*env*	
QB210	17–163 [90]	A	D	D	C†	C†	D	Recombination
QC369	29–143 [86]	A	A	A	A	A	A	Replacement
QD149	996–1086 [1041]	A	A	A	-	-	A	Recombination
QD151	241–801 [521]	A	A	A	A	A	A	Recombination
QD696	49–174 [112]	A	A	A	-	A	A	Recombination
QF441	255–444 [350]	A	A	A	D†	D†	A	Recombination
QF564	17–1270 [644]	A	A	A	D†	D†	A	Recombination
QG262	59–144 [102]	A	A	A	A	A	A	Recombination
QG284	155–260 [208]	A	A	A	A	A	A	Recombination
Proportion subtype A (%)		100.0	88.9	88.9	57.1	62.5	88.9	
Proportion subtype D (%)		0.0	11.1	11.1	28.6	25.0	11.1	
Proportion subtype C (%)		0.0	0.0	0.0	14.3	12.5	0.0	
Proportion intersubtype superinfections (%)					42.8	37.5	0.0	

-superinfecting variant not detected.

† intersubtype superinfection.

**Figure 4:**
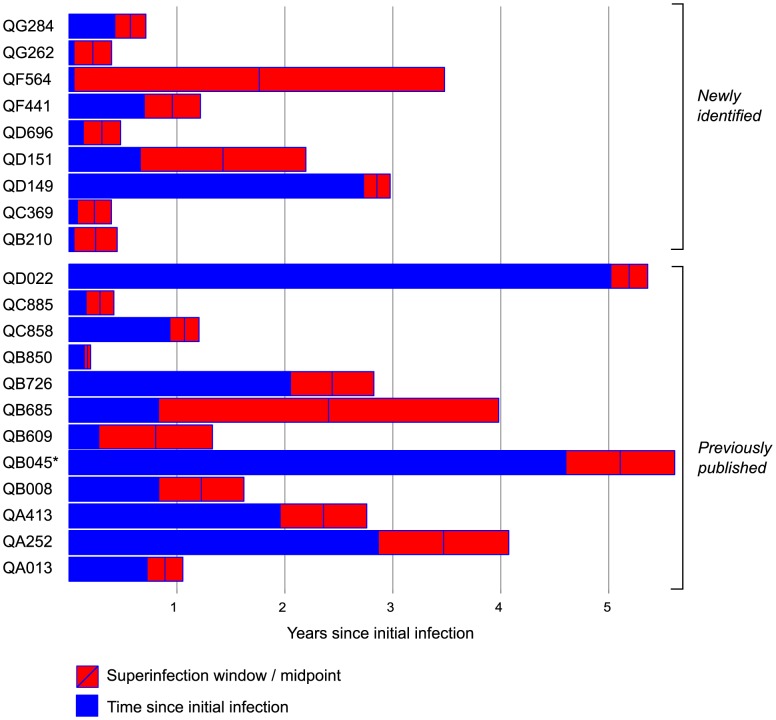
Summary of timing of superinfection events relative to initial infection events. Subject identifiers for the 21 cases of superinfection are listed with the 9 cases identified here listed first, followed by the 12 cases from prior studies [5], [12], [17]. Time since initial infection (years) is represented as blue bars. The red rectangles represent the interval between the last timepoint at which only the initial variant was detected and the first timepoint at which the superinfecting variant was detected. The blue line marks the interval midpoint. *QB045 was HIV RNA-positive at enrollment and was therefore excluded from the incidence analysis.

Combining the data here with those from previous studies in the Mombasa cohort [5], [12], [17], a total of 146 women were examined for superinfection: 90 were tested using NGS, 39 using both NGS and Sanger sequencing, and 17 using only Sanger sequencing. Among the 39 women previously identified as singly infected by Sanger sequencing and tested by NGS here, no new cases of superinfection were identified, suggesting older methods were sensitive enough to detect superinfection. Twenty-one cases of superinfection were confirmed based on detection of the superinfecting virus in two or more samples. The timing windows of all 21 superinfection events are summarized in Figure 4 and Table S2. The midpoint of the timing window of the 9 new cases ranged from 81 to 1041 dpi, with 6 occurring within the first year of infection. The window of superinfection events was defined to a median of within 127 days, with window sizes of 90 to 1253 days. Timing of all 21 cases ranged from 63 to 1895 dpi, defined to a median of within 146 days.

### Sequence similarity between initial and superinfecting viruses

We detected both inter-subtype and intra-subtype superinfections. In 6 of 9 cases identified by NGS, the superinfecting variant was the same subtype as the initial variant in every gene where both were detected. In all 9 cases, the variants were the same subtype in the *env* amplicon (Table 1). Among all 21 cases of superinfection (Table S2), the majority of superinfection events we detected were intrasubtype, regardless of genomic region: 53.8% were intrasubtype based on *gag* sequence, 62.5% based on *pol*, and 70.6% based on *env*.

**Figure 5:**
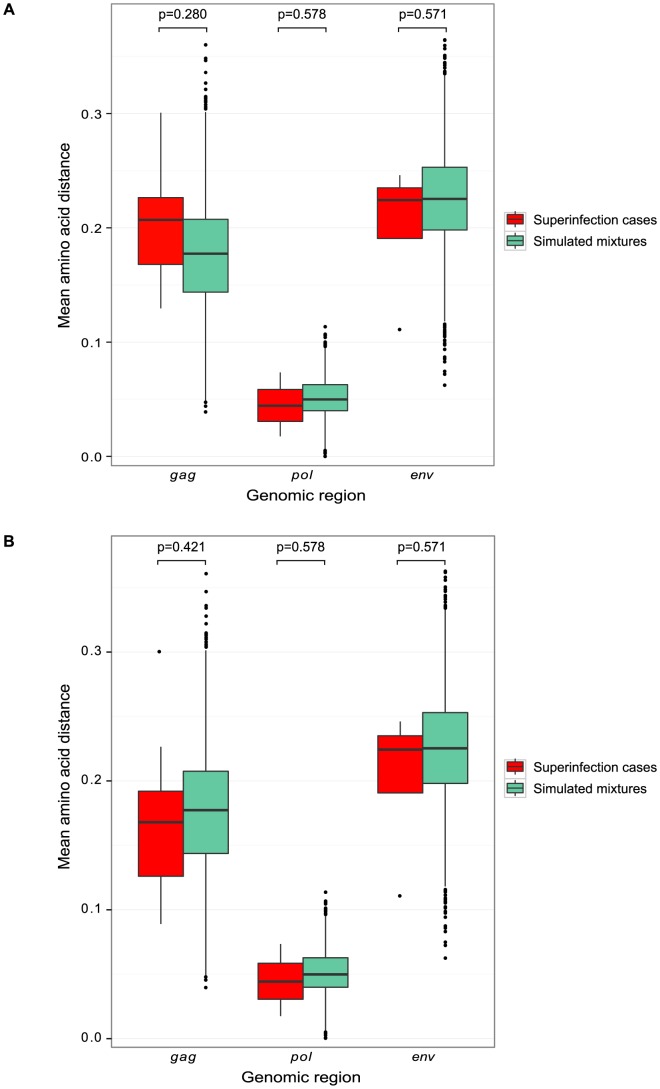
Sequence similarity between initial and superinfecting variants. Boxplots of pairwise amino acid distances in each genomic region are displayed, comparing the distance between the initial and superinfecting sequences within each superinfection case (red) to the distance between sequences in simulated mixtures of randomly selected pairs of singly-infected individuals (green). **A.** Analysis of 454 sequences from NGS screen. **B.** Analysis of 454 sequences from NGS screen and Sanger sequences from 12 previously identified cases of superinfection [5], [12], [17].

We further investigated the possibility of a bias in sequence similarity of superinfecting variants to initial variants by analyzing amino acid diversity. We compared the pairwise amino acid distance between initial and superinfecting variants within each superinfection case to the distance that would be expected by chance. The latter was modeled by simulated mixtures of sequences from all possible pairs of singly infected individuals in the Mombasa cohort (Fig. 5). Using NGS data from the 9 superinfection cases and 120 singly infected women screened here, we found no significant differences between the sequence similarity within superinfected individuals and that expected by chance (Fig. 5a). Including Sanger sequencing data from the additional 12 superinfected women previously screened yielded a similar result (Fig. 5b)

### Incidence and timing of initial infection and superinfection

The incidence of superinfection among women who were screened was compared to the incidence of initial infection in the entire cohort at risk. Only incident HIV infections (occurring after enrollment in the cohort) were included. Fourteen women who were seronegative but HIV RNA positive at enrollment were excluded for this reason. Seven of these had been screened for superinfection, and one was found to be superinfected, which mirrors the frequency of superinfection observed in the entire group. The individual with evidence of dual infection at the earliest timepoint was also excluded, since we were unable to distinguish coinfection from superinfection. After exclusions, 1910 women were at risk of initial infection, contributing 5124 person-years, and 138 women were screened for superinfection, contributing 764py following first infection. There were 295 initial infections, giving a crude incidence rate of 5.7 per 100pys, and 20 superinfections, giving a crude incidence rate of 2.61 per 100 pys.

**Figure 6:**
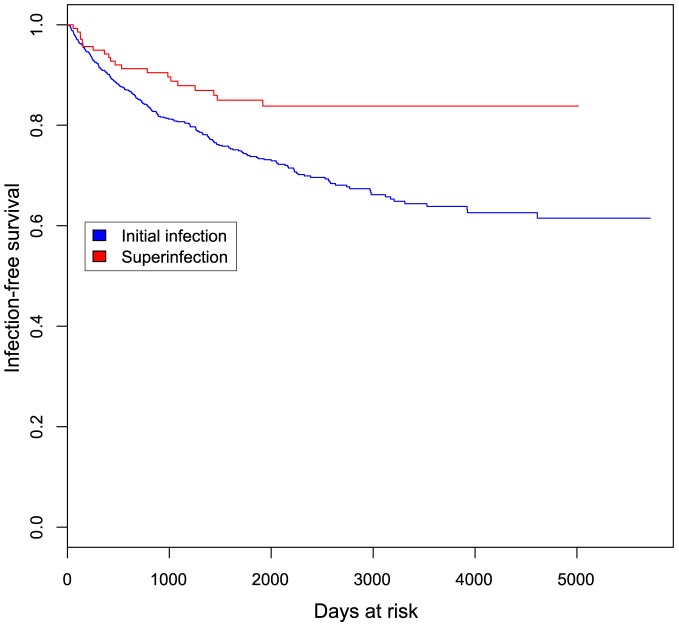
Kaplan-Meier curve showing initial and superinfection events over time at risk. Event-free survival is plotted against days at risk for 1910 women at risk for initial infection (blue) and 138 women at risk for superinfection (red).

The incidence of superinfection and initial infection over time is summarized in Figure 6. We used Andersen-Gill proportional hazards analysis to generate a hazard ratio (HR) relating the incidence of superinfection to that of initial infection. The unadjusted HR for this comparison was 0.49 (CI 0.31–0.76, p = 0.0018). Variables previously shown to influence HIV exposure risk in this cohort [22], [23] were included as adjustments in the model (summarized in Table 2). These included self-reported sexual risk behavior, place of work, hormonal contraceptive use, genital tract infections, years in sexwork, age at first sex, total follow-up time in the cohort and calendar year. The HR for superinfection compared to initial infection, adjusted for these variables, was 0.47 (CI 0.29–0.75, p = 0.0019). Since proportional hazards analysis is based on time to infection and the precision with which superinfection timing was determined varied between cases, we performed sensitivity analyses setting infection timing for all cases to the start or midpoint of the timing windows rather than the end, as done for the above analysis. In both of these analyses, significant differences in incidence were also observed: setting infection timing to the start of the windows, the adjusted HR was 0.33 (CI 0.18–0.58, p = 0.00012); using the window midpoints, the adjusted HR was 0.39 (CI 0.23–0.63, p = 0.00016).

**Table 2:** Sociodemographic and clinical characteristics of women at risk of initial infection and screened for superinfection.

	Initial infection risk set n = 1910	Superinfection risk set (screened) n = 138
Age	26.0 (23.0–21.0)	28 (24.5–33.4)
Years education	8 (7–10)	7 (7–10)†
Parity	1 (1–2)	2 (1–2)†
Bar worker, no. (%)	1410 (74)	117 (85)†
Alcohol, no. (%)	1459 (76)	116 (84)†
Years in sexwork	1.0 (0.1–3.0)	3.2 (1.5–5.7)
Age at first sex	17 (15–18)	17 (15–18)
Sexual risk behavior in past week		
Sex frequency	2 (1–3)	1 (0–2)
Unprotected sex frequency	0 (0–1)	0 (0–1)
Number sexual partners	2 (1–3)	1 (0–1)
Hormonal contraception in past 70 days, no. (%)		
OCP	234 (12)	20 (14)
Depot	400 (21)	53 (38)
Norplant	39 (2)	2 (1)
IUD	41 (2)	3 (2)
STI in past 70 days, no. (%)		
Bacterial vaginosis	665 (35)	71 (51)
Cervicitis	235 (12)	30 (22)
Genital ulcer disease	35 (2)	10 (7)
Gonorrhea	85 (4)	16 (12)
Trichomoniasis	105 (5)	22 (16)
Any	900 (47)	95 (69)
Total years follow-up∧	1.6 (0.3–5.3)	6.7 (4.3–9.8)
Calendar year, median (IQR)	1997 (1995–2002)	1997 (1995–1999)

Values shown are median (IQR), unless otherwise specified.

Data shown were collected at time of entry into each risk set (seroincident cohort enrollment for initial infection risk and initial HIV infection for superinfection risk), except where marked.

† at seroincident cohort enrollment.

∧ Time from seroincident cohort enrollment to censoring, regardless of infection events.

We assessed whether the risk of superinfection varied with time since initial infection by dividing our data into infection events occurring early or late in follow-up and estimating the HR, as above, in each subset. We found that within the first 6 months at risk, the incidence rates of initial and superinfection did not differ significantly (adjusted HR 0.73, p = 0.51), whereas after 6 months the rate of superinfection was lower than that of initial infection (adjusted HR 0.40, p = 0.0017). A similar result was observed when considering events within or beyond one year at risk: within the first year, the incidence rates of initial and superinfection did not differ significantly (adjusted HR 0.54, p = 0.14), but after one year the rate of superinfection was significantly lower (adjusted HR 0.43, p = 0.0059). Sensitivity analyses setting infection time to the start and midpoint of the timing windows as above reproduced the same results (data not shown).

We noted that the previous screens in the cohort appeared to detect a higher frequency of superinfection than the NGS screen (12 cases of 56 women screened, compared with 9 cases of 90), with a greater fraction of the events occurring later after initial infection (Fig. 4). Since the NGS screen spanned later years in the cohort than the previous studies, such a difference could be due to the known decline in infection risk in the cohort over calendar time [22], [23]. However, the numbers of events are small when the datasets are considered separately and the difference both in superinfection incidence rate and post-infection timing between the two studies was not statistically significant (data not shown).

## Discussion

In this study we used NGS to screen for superinfection in 129 high-risk women and identified 9 cases of superinfection. Combined with previous studies[5], [12], [17], a total of 21 cases of superinfection were detected among 146 women screened in this cohort. There was a statistically significant difference between the incidence of superinfection (2.61 per 100pys) and initial infection (5.75 per 100 pys), with a hazard ratio of 0.47 after adjusting for potential confounding factors. This suggests that HIV infection provides partial protection from subsequent infection.

The relatively large size of this cohort and high number of superinfection cases enabled us to detect for the first time a statistically significant difference between the incidence of initial infection and superinfection. This possibility has been proposed previously, though the studies were not designed and/or powered to detect a difference [17], [18]. In the largest incidence study prior to the present study, Redd *et al.* screened a comparable number of individuals (149) in a lower-risk cohort and identified 7 cases of superinfection. The incidence of superinfection was not found to differ significantly from initial infection, but there was a trend for lower incidence of superinfection when controlling for baseline sociodemographic differences between the groups at risk of initial and superinfection. Analysis of our data using the same methods as Redd *et al.* – Poisson regression with propensity score matching [8] – was consistent with the results of our Andersen-Gill analysis, showing a significant difference in incidence, with an estimated incidence ratio of 0.48 (p = 0.011) comparing superinfection to initial infection.

In addition to sample size, two strengths of our incidence analysis were our specification of infection timing to within a few months on average and our comparison of initial and superinfection risk within the same cohort. These enabled us to adjust for the same potential confounding factors in both the initial infection and the superinfection risk sets, using frequently collected time-varying covariate data. Particularly important, given the sequential nature of superinfection, was adjustment for calendar year to control for decline in infection risk in the cohort over time. The distributions of initial and superinfection events over calendar time were similar (Fig. S3), suggesting community-level changes over time did not severely bias our analysis.

The ∼two-fold reduction we found in the incidence of superinfection has a number of possible interpretations. First, it may indicate that the adaptive immune response elicited by initial infection provides partial protection from second infection. If this were the case, superinfection might preferentially occur early in infection, before the response has matured [2], [13], [24]. In support of this idea, we found that, although superinfection occurred throughout the course of first infection, the incidence of superinfection was significantly lower than initial infection after the first 6 months of infection, but not earlier. This suggests that susceptibility to superinfection decreased over time, coincident with broadening and strengthening of HIV-specific immunity. Indeed, this has been suggested by two earlier studies, each documenting three cases of superinfection that occurred within the first year after initial infection [3], [18].

If the difference in incidence we observed is due to a partially protective adaptive immune response, we would anticipate superinfection would preferentially occur with more distantly related viruses, more likely to escape the response. Using viral subtype and pairwise amino acid distance as surrogate measures of antigenic distance, our data provided no evidence of this effect. The majority of the 21 superinfection events we detected were intrasubtype, and the proportion of subtype A, C and D viral sequences was similar for the initial and superinfecting viruses, consistent with the subtype distribution in this cohort [25]. The pairwise distance between initial and superinfecting variants was no higher than the distribution of distances between random pairs of singly-infected individuals from the Mombasa cohort. This may potentially be explained by limited sample size or insufficient simultaneously circulating subtypes. It also may be that sequence relatedness is a poor indicator of susceptibility to the immune response or the genome regions we analyzed are not critical antigenic determinants of protection.

Alternatively, it is possible that protective immune responses are not driving the protective effect we observed. Another potential explanation for the lower risk of superinfection is that HIV infection itself may reduce infection risk by depleting permissive target cells. On the other hand, chronic immune activation and immunodeficiency following HIV infection could increase susceptibility, potentially blunting protective effects [26]. Thus, there may be a complex interplay of biological factors impacting HIV risk in an HIV-positive individual.

So far, studies of immune correlates of superinfection have yielded variable results – some suggesting neutralizing antibody deficits in superinfection [27], [28], while others, including studies in the Mombasa cohort, detected no differences in antibody [29], [30] or cellular [31] responses. A major challenge in these studies has been the identification and analysis of large enough numbers of superinfection cases: the small sample sizes in studies to date (three to twelve superinfected individuals) would restrict detection to only very large effects. Small sample size is just one factor that has made detecting immune deficits associated with superinfection challenging and contributed to variable results among studies. There has also been variation among published studies in the control groups used for comparison, including the time at which the response was analyzed relative to the time of superinfection and initial infection. Given the dynamic nature of the immune response, sample timing could impact measures in both controls and cases. Furthermore, precision in the estimated timing of superinfection varies between studies, and between cases, providing an additional variable. Divergent findings between studies may also reflect differences in the assays used and subtleties in the immune parameters they capture.

Our finding of lower risk of superinfection than initial infection provides greater impetus for larger-scale comprehensive analysis of multiple immune mechanisms, including both those analyzed in the smaller studies to date and, perhaps of more interest, those not characterized in prior studies. If the discrepancies in earlier studies reflect the fact that multiple immune parameters are at play, then examining a variety of immune responses in the same individuals in a larger cohort may be needed to define responses that contribute to HIV susceptibility following initial infection.

Like all studies, the study presented here has a number of limitations. Firstly, while our screening methods are among the most sensitive developed, it remains possible that some cases of superinfection were missed. In particular, reinfection by the same source partner is not captured by any existing methods. Additionally, our specification of the timing of superinfection was limited by the samples available to us. While follow-up was generally frequent in this study population, there were six superinfection cases where sample availability limited our ability to define the time of superinfection to within a one-year period. This uncertainty in superinfection timing did not affect our findings, as we found that whether we assumed in the incidence analysis that the true timing of superinfection was at the start, midpoint or end of the timing window, the results indicated that the incidence of superinfection was significantly lower than that of initial infection. Finally, as in all observational studies, residual confounding of our incidence estimate by behavioral changes and sexual network-level factors not measured or accounted for in our analyses remains a possibility. However, the fact that we compared initial and superinfection risk within the same cohort and collected covariate data at frequent intervals enabled us to minimize this issue to an extent not possible in previous studies.

This study provides the first robust evidence that HIV infection reduces the risk of subsequent infection. The underlying mechanism remains unclear, but this finding prompts exploration of correlates of protection from HIV in high-risk individuals who continue to be exposed after first infection. Furthermore, this study reinforces that superinfection occurs at a considerable rate, calling for studies of its impact on the clinical progression, transmission, and epidemiology of HIV.

## Materials and Methods

### Ethics statement

The study was approved by the ethical review committees of the University of Nairobi, the University of Washington and the Fred Hutchinson Cancer Research Center. Written informed consent was obtained from all participants.

### Study population

Seronegative women in Mombasa, Kenya, attended monthly visits, at which clinical examinations, interviews and sample collection took place, as previously described [22]. Following seroconversion, sample collection took place quarterly. Individuals were selected for superinfection screening based on sample availability <6 months and >2 years post-initial HIV infection, and an approximately equally spaced intervening sample. Within these limitations, samples with maximal plasma viral load, >1000 copies/ml, and prior to initiation of antiretroviral therapy were selected. Thirty-nine of 44 women previously screened for superinfection by Sanger sequencing and identified as singly infected [5], [12], [17] were rescreened; the remaining 5 women did not have adequate samples available.

### Viral amplification and sequencing

HIV virions were isolated from heparinized plasma using the μMACS VitalVirus HIV Isolation kit (Miltenyi Biotec) and viral RNA extracted from 140–420 µl, depending on viral load, using the Qiamp viral RNA Mini kit (Qiagen). Nested RT-PCR of ∼500 bp in *gag*, *pol* and *env* was conducted in duplicate (see Table S1). RNA input into each reaction was normalized to 3000 viral genomes according to plasma viral load, or the maximum possible where viral load was too low. RT-PCRs for the three genes were multiplexed. Nested PCR reactions were carried out separately for each region with primers containing adaptors for Roche 454 sequencing and a unique 8 bp barcode sequence to identify each sample. PCR products were purified using AMPure XP PCR purification beads (Agencourt) and quantified using the Qubit dsDNA HS assay (Invitrogen). PCR products were sequenced on the Roche 454 GS-Junior or GS-FLX titanium platform. Where initial sequencing suggested superinfection (see below), timing was inferred by sequencing intervening timepoints. Sequences are available upon request from the authors.

### Bioinformatic pipeline

454 sequences were error-corrected using AmpliconNoise [32]. Chimeric sequences were identified and removed using UCHIME [33]. Cross-contamination between samples sequenced together and contamination by other lab samples was identified by all-against-all BLAST against a local database of published HIV sequences and sequences from the same sequencing run. Sequences with high identity hits to known laboratory stains or other samples from the same sequencing run were removed. Sequences with abundance <5 reads or 0.5% of the sample, whichever was higher, were excluded from further analyses as lower abundance variants were not reproducibly detected in repeated deeper sequencing of two selected samples where rare variants formed a distinct phylogenetic clade. An amplicon-specific profile HMM was created from an alignment of representative sequences from multiple subtypes. For each subject and amplicon, 20 reference sequences were selected by placing 454 reads on a tree of candidate reference sequences [34] and minimizing the average distance to the closest leaf [35]. These reference sequences, representatives from subtypes common to the region, and 454 reads were aligned to the HMM using hmmalign [36] and non-consensus columns removed. Any sequences <200 bp long after alignment and trimming were removed. We used BEAST [37] to calculate a posterior probability of monophyly for the sequences. A posterior sample of trees was obtained using a strict molecular clock, Bayesian Skyline Plot population model and the HKY substitution model. Each MCMC chain ran 20 million iterations, sampling every 2000, discarding the initial 25% of samples as burn-in. Chains were assessed for convergence by examining effective sample size (ESS) and by visual inspection of traces of key parameters. A strict clock was used as poor mixing was frequently observed under relaxed clock models. BEAST runs with intermediate posterior probabilities (0.2–0.8) were manually examined for recombinant sequences and run again with putative recombinants removed. Pairwise distances were calculated for all sequence pairs under the TN93 model using APE [38], reporting the maximum within-subject distance. For comparison, 95% confidence limits of pairwise distances were calculated for sequences from known single infections (previously screened in [5], [12], [17]) and simulated dual infections. Dual infections were simulated by combining all pairs of sequences from previously screened singly infected samples. Pairwise distances calculated from 454 sequences obtained in this study were compared to the upper bound of the 95% quantile of single infection distances, and the lower bound of the 95% quantile of simulated dual infection distances. This pipeline was validated and refined by processing monophyletic viral isolates, known mixtures of isolates, and known cases of superinfection detected by Sanger sequencing [17]. These methods were found to be sensitive enough to distinguish two subtype A isolates mixed at abundances of 5%∶95% genome copies in all three genomic regions, and at 1%∶99% in two of three genomic regions (Fig. S1).

### Analysis of amino acid distance in superinfection

Sequences were aligned as for the phylogenetic analysis. Insertions relative to the reference alignment were removed, and sequences with <60% coverage or identified as recombinants between initial and superinfecting variants upon visual inspection were excluded. For each case of superinfection, viral sequences were annotated as the initial strain or the superinfecting strain. We calculated the mean Hamming distance between amino acid sequences of the superinfecting strain from the time of superinfection detection and sequences of the initial strain up to and including this time. In calculating the mean distance, each pairwise comparison was weighted using the product of the multiplicities of the two reads. To investigate whether these distances deviated from what would be expected by chance, an artificial set of mock superinfections was generated by combining sequences from singly infected individuals. All pairs of singly infected individuals screened by 454 sequencing were enumerated. In each pair, one individual was randomly chosen to be the source of the ‘initial’ virus in the simulated superinfection. A time of ‘superinfection’ was chosen randomly from the available sampled timepoints and sequences from all timepoints up to and including this time were used for analysis. The other individual in the pair acted as the source of the ‘superinfecting’ virus. A time of ‘transmission’ was chosen randomly from the available sampled timepoints and sequences from this timepoint were used. Mean distances within pairs were calculated as above. The analysis was repeated including *gag* and *env* Sanger sequences from previously published cases [5], [12], [17], trimmed to the genome region amplified for NGS, and given unit weight. A two-sample Wilcoxon test was used to test for a difference between the distances observed in true superinfections and those simulated in mock superinfections.

### Incidence analysis

Statistical analysis was performed using R (www.r-project.org). The incidences of initial and superinfection were compared by Andersen-Gill proportional hazards analysis. The predictor was inclusion in the screen for superinfection, modeled as a time-dependent variable, and the outcome was time to HIV infection (initial and super). Timing of infection events for the incidence analysis was set to the study visit of their detection (for initial infection events the visit after inferred infection timing; for superinfection events, the time at which the superinfecting virus was first detected). Individuals who were HIV infected but not screened for superinfection were censored after acquisition of initial infection. Individuals who became superinfected were censored after acquisition of superinfection. Individuals who were screened and not found to be superinfected were censored at the last timepoint screened. Since samples after initiation of antiretroviral treatment were excluded from superinfection screening, no follow-up after treatment initiation was included. The model was adjusted for time-varying variables at each visit: calendar year, age, years in sexwork, number of weekly sexual partners, number of weekly unprotected sex acts, hormonal contraceptive use in the prior 70 days and any genital tract infection in the prior 70 days (bacterial vaginosis, cervicitis, genital ulcer disease, gonorrhea, trichomoniasis); place of work and age at first sex recorded at enrollment; and total follow-up time in the study. Incidences of initial and superinfection were also estimated as described in [8], using Poisson regression and propensity score matching to select a subset of women at risk of initial infection whose baseline risk profiles most closely matched those of women screened for superinfection.

## Supporting Information

Figure S1 **Detection of control viral mixtures at 95∶5 & 99∶1.** Phylogenetic trees representing viral sequences in *gag*, *pol* and *env* from known mixtures of plasma from two individuals (QA966 and QF927) at ratios of 95∶5 and 99∶1.
(PDF)

Figure S2 **Schematics of viral variants detected in 9 cases of superinfection.** Detection of initial and superinfecting variants is indicated in each of the three genomic regions at each timepoint.
(PDF)

Figure S3 **Calendar year at the estimated time of initial infection and superinfection in superinfection cases.**
(PDF)

Table S1 **PCR primers and conditions.**
(XLSX)

Table S2 **Characteristics of all 21 cases of superinfection.**
(XLSX)
