# Supplementary figures and images for: HIV-1 Superinfection Occurs Less Frequently Than Initial Infection in a Cohort of High-Risk Kenyan Women
Source: PLoS Pathog. 2013 Aug 29;9(8):e1003593. doi: 10.1371/journal.ppat.1003593 (PMC3757054; doi:10.1371/journal.ppat.1003593)

# Known mixture1: 95% QA966 5% QF927

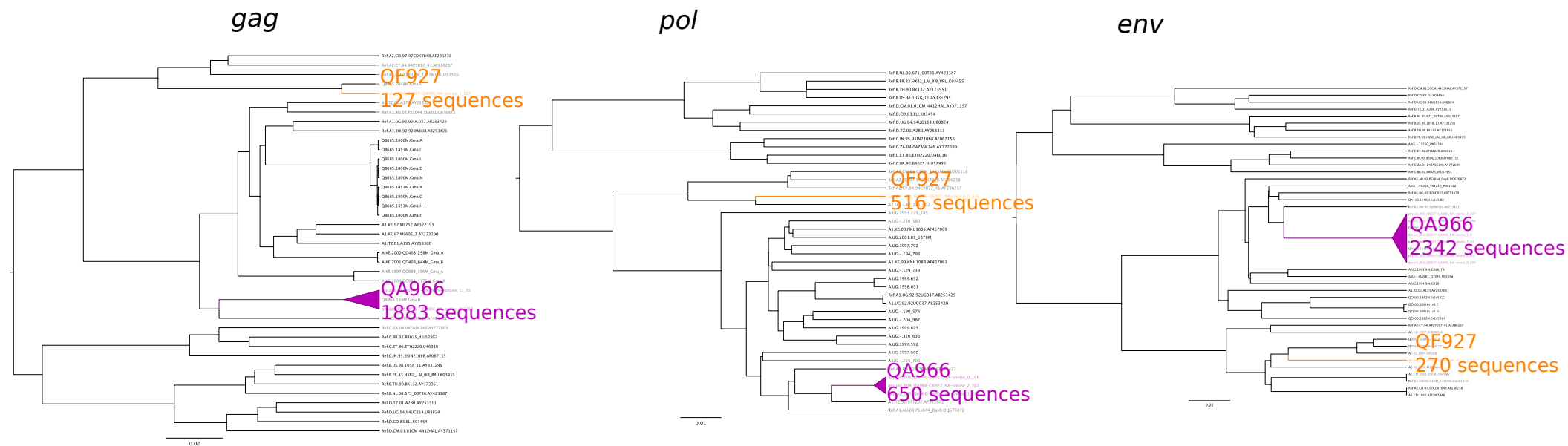

# Known mixture2: 99% QA966 1% QF927

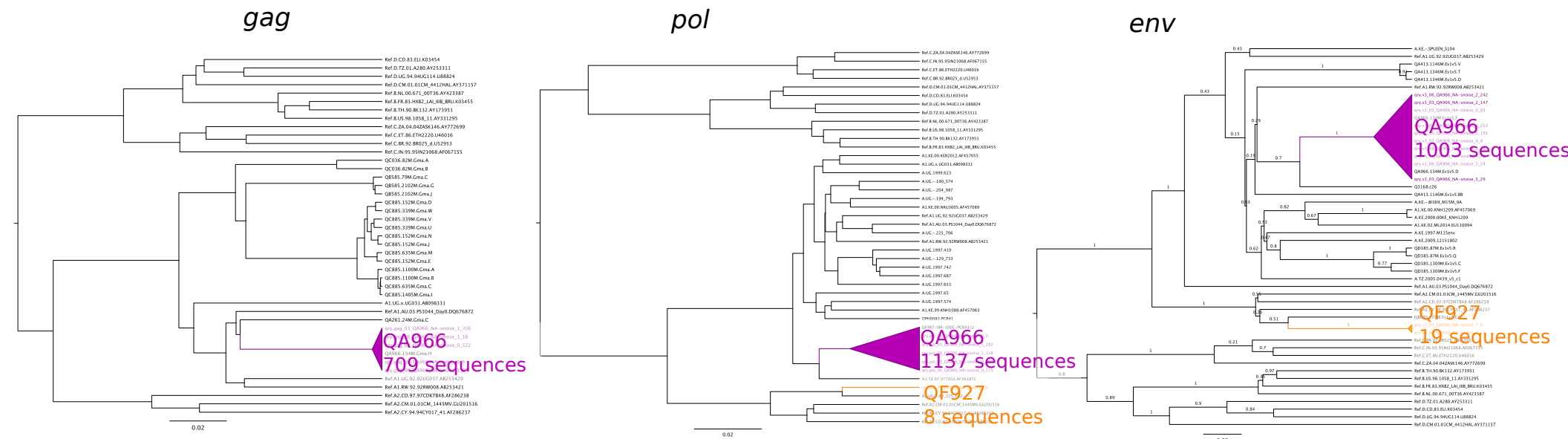

Supplement: Figure S1 — Detection of control viral mixtures at 95∶5 & 99∶1. Phylogenetic trees representing viral sequences in gag, pol and env from known mixtures of plasma from two individuals (QA966 and QF927) at ratios of 95∶5 and 99∶1. (PDF) [file ppat.1003593.s001.pdf]

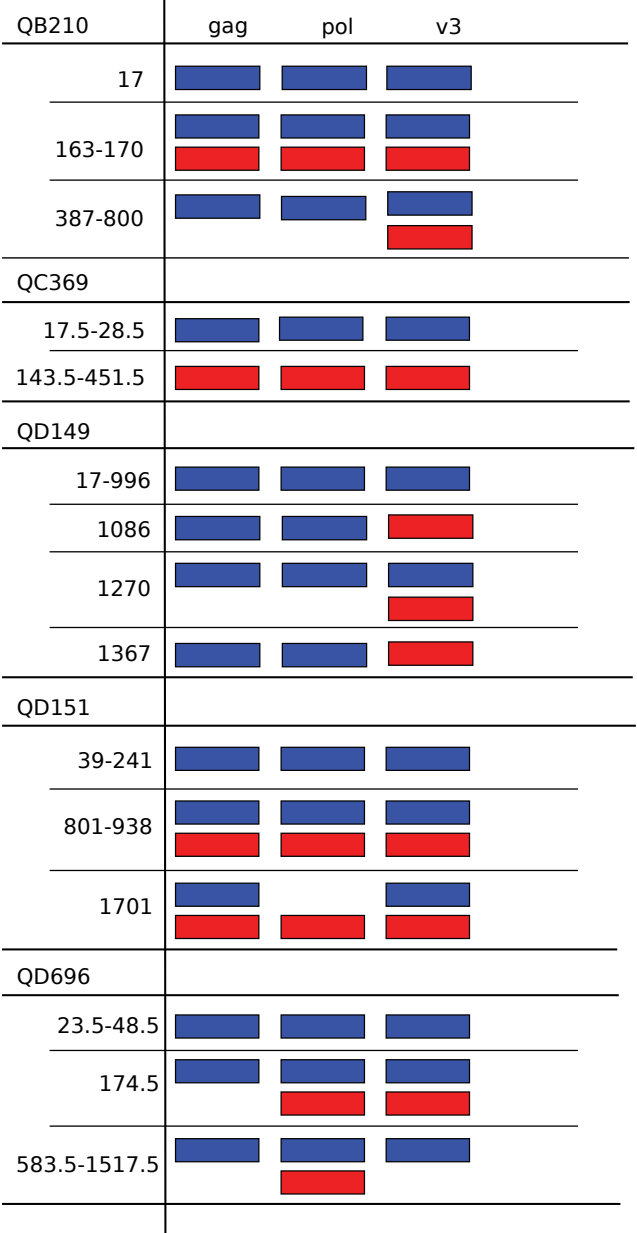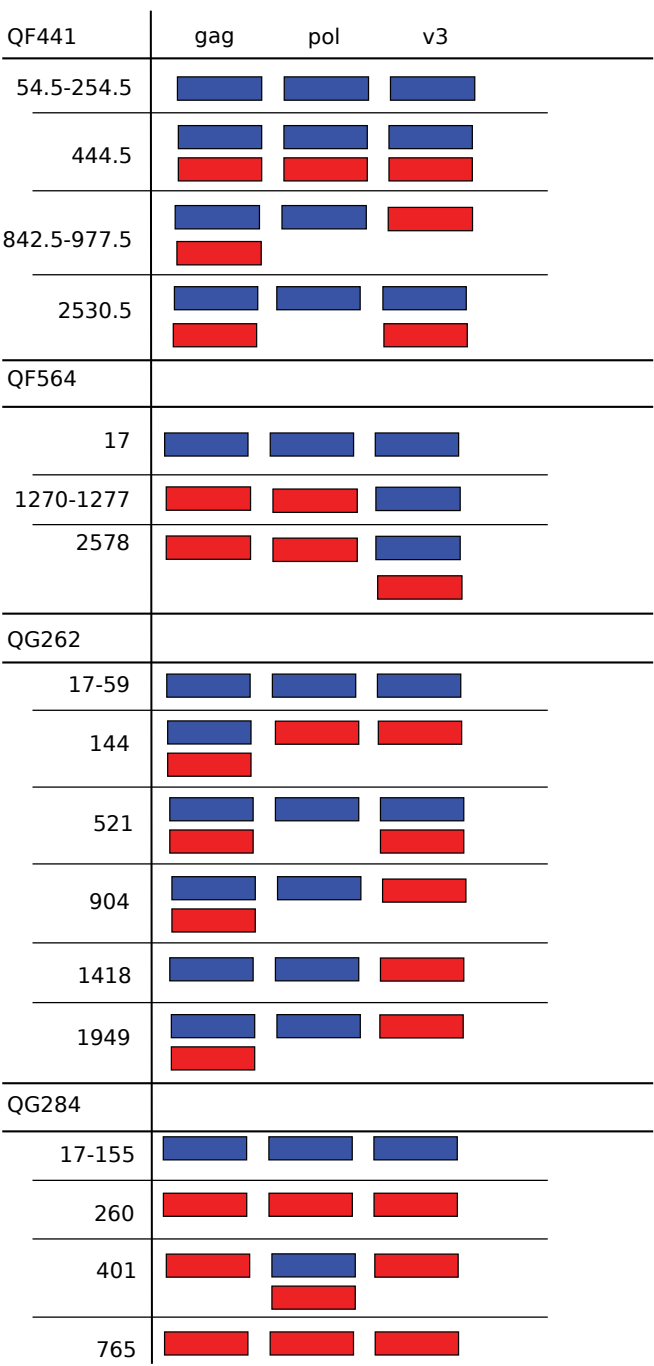

Initial variant

Superinfecting variant

Supplement: Figure S2 — Schematics of viral variants detected in 9 cases of superinfection. Detection of initial and superinfecting variants is indicated in each of the three genomic regions at each timepoint. (PDF) [file ppat.1003593.s002.pdf]

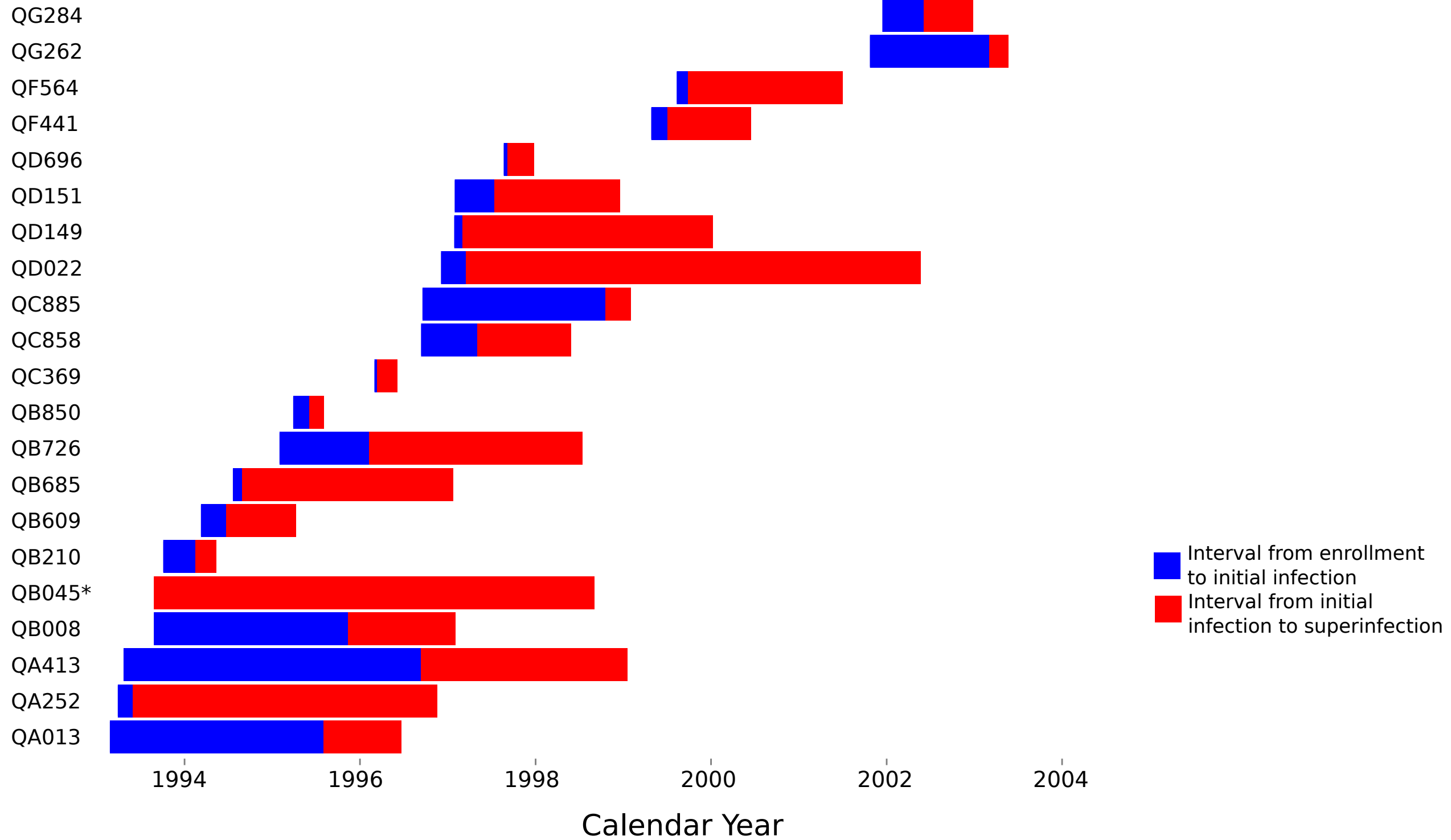

Supplement: Figure S3 — Calendar year at the estimated time of initial infection and superinfection in superinfection cases. (PDF) [file ppat.1003593.s003.pdf]
